# The effects of muscle mass and quality on mortality of patients with acute kidney injury requiring continuous renal replacement therapy

**DOI:** 10.1038/s41598-023-33716-9

**Published:** 2023-05-05

**Authors:** Jiyun Jung, Jangwook Lee, Jeong-Hoon Lim, Yong Chul Kim, Tae Hyun Ban, Woo Yeong Park, Kyeong Min Kim, Kipyo Kim, Sung Woo Lee, Sung Joon Shin, Seung Seok Han, Dong Ki Kim, Yousun Ko, Kyung Won Kim, Hyosang Kim, Jae Yoon Park

**Affiliations:** 1 Clinical Trial Center, Dongguk University Ilsan Hospital, Goyang, South Korea; 2 Research Center for Chronic Disease and Environmental Medicine, Dongguk University College of Medicine, Gyeongju, South Korea; 3 Department of Internal Medicine, Dongguk University Ilsan Hospital, Goyang, South Korea; 4 Department of Internal Medicine, Kyungpook National University Chilgok Hospital, School of Medicine, Kyungpook National University, Daegu, South Korea; 5 Department of Internal Medicine, Seoul National University Hospital, Seoul, South Korea; 6 Department of Internal Medicine, Eunpyeong St. Mary’s Hospital, College of Medicine, The Catholic University of Korea, Seoul, South Korea; 7 Department of Internal Medicine, Keimyung University Dongsan Hospital, Keimyung University School of Medicine, Daegu, South Korea; 8 Department of Internal Medicine, Daejeon Eulji Medical Center, Eulji University, Daejeon, South Korea; 9 Department of Internal Medicine, Inha University Hospital, Inha University College of Medicine, Incheon, South Korea; 10 Department of Internal Medicine, Uijeongbu Eulji Medical Center, Eulji University, Gyeonggi-Do, South Korea; 11 Department of Internal Medicine, Dongguk University College of Medicine, Gyeongju, South Korea; 12 Biomedical Research Center, Asan Institute for Life Sciences, Asan Medical Center, Seoul, South Korea; 13 Department of Radiology, Asan Medical Center, University of Ulsan College of Medicine, Seoul, South Korea; 14 Division of Nephrology, Department of Internal Medicine, Asan Medical Center, University of Ulsan College of Medicine, Seoul, South Korea

**Keywords:** Nephrology, Kidney diseases

## Abstract

This study examined the effects of muscle mass on mortality in patients with acute kidney injury requiring continuous renal replacement therapy. It was conducted in eight medical centers between 2006 and 2021. The data of 2200 patients over the age of 18 years with acute kidney injury who required continuous renal replacement therapy were retrospectively collected. Skeletal muscle areas, categorized into normal and low attenuation muscle areas, were obtained from computed tomography images at the level of the third lumbar vertebra. Cox proportional hazards models were used to investigate the association between mortality within 1, 3, and 30 days and skeletal muscle index. Sixty percent of patients were male, and the 30-day mortality rate was 52%. Increased skeletal muscle areas/body mass index was associated with decreased mortality risk. We also identified a 26% decreased risk of low attenuation muscle area/body mass index on mortality. We established that muscle mass had protective effects on the mortality of patients with acute kidney injury requiring continuous renal replacement therapy. This study showed that muscle mass is a significant determinant of mortality, even if the density is low.

## Introduction

Acute kidney injury (AKI) is a common complication in critically ill patients during hospitalization^1^ and a significant independent risk factor for patient survival and progression to chronic kidney disease (CKD)^2^. Continuous renal replacement therapy (CRRT) is a method of resolving the imbalance of metabolites and electrolytes without causing rapid hemodynamic and biochemical fluctuations; it plays a vital role in treating critically ill patients with severe AKI^3^. However, despite advances in critical care medicine over the past decades, the mortality rate of patients with severe AKI who undergo CRRT is reported to be 50–80%^4,5^. Thus, it is necessary to identify new factors to predict and reduce mortality risk in patients with severe AKI and progression to CKD in survivors after treatment. Since its first introduction by Irwin Rosenberg in 1989, sarcopenia has been used as a diagnostic tool for frailty^6,7^. Sarcopenia can cause obesity by reducing the basal metabolic rate and was found to be a risk factor for dysphagia and falls and a significant risk factor for mortality in critically ill patients^6,8,9^. The Asian Working Group for Sarcopenia (AWGS) defined the diagnostic tools for sarcopenia as muscle strength, physical performance, and appendicular skeletal muscle mass in clinical research settings for Asians^10^. However, applying this method properly to critically ill patients who are bedridden and have difficulty in functional evaluation is difficult. The artificial intelligence (AI)-based diagnostic evaluation of sarcopenia using imaging data can be a standardized diagnostic tool with high accuracy^11,12^. It is possible to evaluate the effect of sarcopenia on mortality in critically ill patients who are difficult to evaluate functionally using this technique.

Therefore, we measured the muscle mass of patients’ abdominal computed tomography (CT) images and evaluated the effect of sarcopenia on the mortality of critically ill patients who underwent CRRT in multiple medical centers in Korea.

## Methods

**Figure 1:**
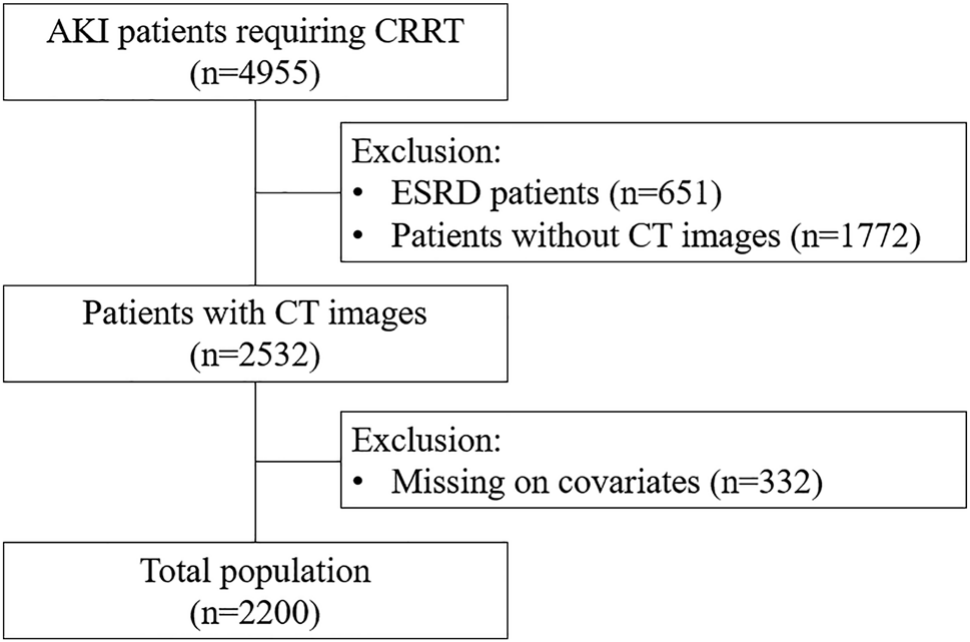
Flow chart of the study population.

In this retropecitve cohort study, data on 4955 patients with AKI aged over 18 years who required CRRT in eight multi-centers between 2006 and 2021 were collected. We excluded patients with end-stage renal disease (n = 635), without abdominal CT images (n = 1772), and with missing information on covariates (n = 332) (Fig. 1). Finally, 2200 patients were enrolled in the study.

Demographic and laboratory information, including sex, age, height (m), weight (kg), body mass index (BMI; kg/m^2^), albumin, hemoglobin, C-reactive protein (CRP), prothrombin time and international normalized ratio (PT INR), Charlson comorbidity index (CCI), CRRT settings (including prescribed dose), sequential organ failure assessment (SOFA) score, and Acute Physiology and Chronic Health Evaluation (APACHE II) score, were retrospectively collected. The prescribed dose was calculated as the sum of dialysate flow rate, replacement flow rate, and fluid removal divided by weight. The CCI considers 19 diseases with different weights to show the severity of comorbidity^13^. The APACHE II is a representative scoring system for patients in the intensive care unit (ICU) using 12 physiological variables; in contrast, SOFA predicts mortality in the ICU according to the severity in six organ systems^14,15^. In addition, we investigated in-hospital mortality within 1, 3, and 30 days.

Standardized non-enhanced abdominal CT scans performed 15 days before or after CRRT initiation were obtained, and slices were selected at the level of the third lumbar vertebra (L3). CT imaging, especially measured at the L3 level, is a representative measurement standard for evaluating body composition^16^. For selecting L3 levels, the YOLOv3-based L3 slice selection algorithm using a deep learning model developed by Asan medical center (AMC) was applied to CT images from AMC, whereas skilled experts selected L3 level slices individually from images in the other medical centers. Automatic selection methods for a single L3 slice from multiple series of abdominal CT images have high accuracy and performance^17^. After selecting a single L3 slice, automated AI software was used to measure the cross-sectional areas of the body composition. Previous studies have validated the deep learning model applied to Asan J in body morphometric analysis^18–20^. It divides the boundary of low attenuation muscle area (LAMA), normal attenuation muscle area (NAMA), intermuscular adipose tissue, visceral fat area, and subcutaneous fat area (cm^2^) using predefined Hounsfield units (HU). To evaluate muscle quality, LAMA and NAMA were measured according to CT density from -29 to 29 HU and from 30 to 150 HU, respectively. The LAMA, reflecting unhealthy muscle, includes intramyocellular lipids, whereas the NAMA, which represents healthy muscle without myosteatosis, reflects little intramuscular fat^12^. The skeletal muscle area (SMA) ranged from -29 to 150 HU, indicating a combined area of LAMA and NAMA. All measurements were divided by the square of height (m^2^), weight (kg), and BMI (kg/m^2^).

Cox proportional hazard models were used to estimate the hazard ratios (HRs) and 95% confidence intervals (CIs) on 1-day, 3-day, and 30-day mortality associated with various skeletal muscle indices: (1) model 1: crude model stratified by CRRT initiation years and medical centers; (2) model 2: additionally adjusted by sex and age; and (3) model 3: fully adjusted model further adjusted by albumin, hemoglobin, PT INR, CRP, history of hypertension, CCI, AKI cause, and prescribed dose of CRRT. A Kaplan–Meier estimation for 30-day mortality associated with quantiles of the muscle index was conducted to compare the survival probability. In addition, we applied muscle index as a continuous and categorical variable in various models. In the categorical model, the HRs in each quartile compared to the lowest quartile as references were estimated, and ordinal values tested linearity for each quartile. The HRs by the interquartile range (IQR) increase in the total patient population are presented in the continuous model. To understand the dose–response curve between 30-day mortality and muscle index, we used a non-linear curve using a penalized spline basis with three degrees of freedom in a fully adjusted model. Moreover, we compared the 1-, 3-, and 30-day mortality risks associated with increased muscle index in the fully adjusted model. We also used area under the curve (AUC) in the Receiver Operating Characteristic (ROC) curve analysis to confirm the predictive ability.

To investigate the susceptible groups, stratified analyses were conducted to determine the association between muscle index as a continuous variable and 30-day mortality by sex, age (< 65 and ≥ 65 years), APACHE II (< median and ≥ median), SOFA (< median and ≥ median), AKI cause (sepsis and non-sepsis), hypertension, and diabetes. All statistical analyses were performed using R version 4.1.1. (R Foundation for Statistical Computing, Vienna, Austria).

## Ethics approval and consent to participate

This study was conducted in agreement with the principles of the Declaration of Helsinki and informed consent was waived by the Institutional Review Boards of Dongguk University Ilsan Hospital (DUIH 2018–12-010–001), Kyungpook National University Chilgok Hospital (KNUCH 2021–03-024), Seoul National University Hospital (H-2111–057-1271), the Catholic University of Korea Eunpyeong St. Mary’s Hospital (PC21RIDI0111), Keimyung University Dongsan Medical Center (DSMC 2021–06-057), Daejeon Eulji Medical Center (EMC 2021–07-006–002), Inha University Hospital (2021–09-029–000), and Asan Medical Center (S2021-1790–0001).

## Results

Table 1 shows the baseline characteristics of 2200 patients, represented as quartiles of skeletal muscle mass adjusted by BMI. The majority of patients (59.9%) were male and were aged over 65 years. Sixty-seven percent of patients had low or normal weight, and sepsis was the most frequent cause of AKI across all quartile groups, accounting for 58.9% of cases. The CCI ranged from 0 to 17, with 86% of patients having at least one comorbidity. Furthermore, the mean APACHE and SOFA scores were 27.3 and 11.4, respectively. In terms of the quartile groups, hypertension and diabetes displayed a trend towards a higher adjusted skeletal muscle mass group, while APACHE and SOFA scores did not vary significantly. CRP levels were significantly lower in the higher adjusted skeletal muscle mass group, whereas hemoglobin, albumin, and PT INR did not exhibit significant differences. The distribution of various muscle measurements is presented in Table 2. The average SMA of all patients was 110.5 cm^2^ and ranged from 28.5 to 237.8 cm^2^. The respective minimum and maximum average SMA were 1.7 cm^2^ and 174.2 cm^2^ for NAMA and 2.9 cm^2^ and 159.2 cm^2^ for LAMA. The mean LAMA was higher than the mean NAMA in all patients and in 30-day deceased patients and survivors.

**Table 1:** Baseline characteristics of the 2200 participants who received continuous renal replacement therapy in multiple medical centers.

Variable	Total (n = 2200)	SMA/BMI				
		Q1 [0.99–3.93]	Q2 [3.93–4.76]	Q3 [4.76–5.58]	Q4 [5.58–9.76]	*p* value
Sex, n (%)						
Male	1318 (59.9)	109 (19.8)	263 (47.8)	441 (80.2)	505 (91.8)	< 0.001
Female	882 (40.1)	441 (80.2)	287 (52.2)	109 (19.8)	45 (8.2)	
Age, n (%)						
18–34	83 (3.8)	19 (3.5)	18 (3.3)	19 (3.5)	27 (4.9)	< 0.001
35–49	220 (10.0)	28 (5.1)	54 (9.8)	69 (12.5)	69 (12.5)	
50–64	570 (25.9)	94 (17.1)	127 (23.1)	153 (27.8)	196 (35.6)	
65–98	1327 (60.3)	409 (74.4)	351 (63.8)	309 (56.2)	258 (46.9)	
BMI, n (%)						
< 25	1465 (66.6)	255 (46.4)	376 (68.4)	399 (72.5)	435 (79.1)	< 0.001
25–29	576 (26.2)	208 (37.8)	136 (24.7)	130 (23.6)	102 (18.5)	
≥ 30	159 (7.2)	87 (15.8)	38 (6.9)	21 (3.8)	13 (2.4)	
AKI cause, n (%)						
Septic	1295 (58.9)	339 (61.6)	350 (63.6)	325 (59.1)	281 (51.1)	< 0.001
Nephrotoxin	93 (4.2)	23 (4.2)	16 (2.9)	28 (5.1)	26 (4.7)	
Ischemia	343 (15.6)	70 (12.7)	74 (13.5)	104 (18.9)	95 (17.3)	
Post-op	162 (7.4)	41 (7.5)	34 (6.2)	35 (6.4)	52 (9.5)	
Volume control	75 (3.4)	20 (3.6)	17 (3.1)	12 (2.2)	26 (4.7)	
Others	232 (10.5)	57 (10.4)	59 (10.7)	46 (8.4)	70 (12.7)	
Comorbidity, n (%)						
Hypertension	838 (38.1)	259 (47.1)	215 (39.1)	195 (35.5)	169 (30.7)	< 0.001
Diabetes	788 (35.8)	219 (39.8)	205 (37.3)	195 (35.5)	169 (30.7)	0.014
Any tumor	793 (36.0)	184 (33.5)	209 (38.0)	225 (40.9)	175 (31.8)	0.006
Metastatic solid tumor	152 (6.9)	42 (7.6)	45 (8.2)	38 (6.9)	27 (4.9)	0.154
Myocardial infarction	195 (8.9)	48 (8.7)	48 (8.7)	56 (10.2)	43 (7.8)	0.582
Congestive heart failure	330 (15.0)	85 (15.5)	78 (14.2)	89 (16.2)	78 (14.2)	0.736
Peripheral vascular disease	175 (8.0)	45 (8.2)	47 (8.5)	39 (7.1)	44 (8.0)	0.834
Peptic ulcer disease	123 (5.6)	39 (7.1)	33 (6.0)	28 (5.1)	23 (4.2)	0.183
Biochemical data, mean (SD)						
Hemoglobin (g/dL)	9.5 (2.2)	9.4 (2.1)	9.4 (2.1)	9.6 (2.4)	9.6 (2.4)	0.148
Albumin, (g/dL)	2.7 (0.7)	2.7 (0.6)	2.6 (0.7)	2.7 (0.6)	2.8 (0.7)	0.004
PT INR	2.7 (4.4)	2.7 (3.9)	3.3 (5.6)	2.5 (3.6)	2.6 (4.2)	0.016
CRP (mg/dL)	12.8 (10.9)	14.2 (11.6	13.2 (10.6)	12.4 (10.9)	11.5 (10.5)	< 0.001
Stay length (day), mean (SD)						
Hospital	40.0 (62.0)	40.3 (70.3)	39.4 (62.2)	41.2 (61.7)	39.2 (53.0)	0.951
ICU	14.7 (28.5)	15.4 (26.4)	13.1 (24.9)	16.4 (36.9)	13.8 (23.7)	0.229
Mechanical ventilation, n (%)	1555 (70.7)	386 (70.1)	390 (71.0)	398 (72.3)	381 (69.3)	0.746
CRRT duration (day), mean (SD)	7.12 (12.5)	6.3 (11.4)	6.5 (13.1)	8.4 (13.5)	7.2 (11.9)	0.028
CRRT setting, mean (SD)						
Prescribed dose (mL/kg/h)	42.5 (19.6)	40.7 (18.9)	44.3 (21.3	43.1 (20.8	41.7 (17.2	0.014
Blood flow rate (mL/min)	114.4 (25.7)	113.8 (26.0)	115.4 (26.9	113.0 (23.9	115.5 (26.1	0.288
Dialysate flow rate (mL/h)	1184.9 (442.2)	1162.6 (449.7)	1205.3 (469.4)	1219.9 (462.0	1151.7 (379.2	0.028
Replacement flow rate (mL/h)	961.7 (584.7)	956.2 (581.8)	949.0 (570.4)	969.2 (610.4	972.4 (576.6	0.901
Charlson comorbidity index, n (%)						
0	305 (13.9)	73 (13.3)	59 (10.7)	71 (12.9)	102 (18.5)	0.001
1–5	1452 (66.0)	360 (65.5)	370 (67.3)	362 (65.8)	360 (65.5)	
6–10	398 (18.1)	104 (18.9)	101 (18.4)	108 (19.6)	85 (15.5)	
11–17	45 (2.0)	13 (2.4)	20 (3.6)	9 (1.6)	3 (0.5)	
APACHE II score, mean (SD)	27.3 (7.9)	27.4 (7.7)	27.7 (7.8)	27.3 (7.6)	26.7 (8.4)	0.261
SOFA score, mean (SD)	11.4 (3.5)	11.1 (3.4)	11.3 (3.3)	11.5 (3.6)	11.1 (3.6)	0.414

*CRRT* Continuous Renal Replacement Therapy; *PT INR* Prothrombin Time and International Normalized Ratio; *APACHE II* Acute Physiology and Chronic Health Evaluation; *SOFA* sequential organ failure assessment; *SMA* Skeletal Muscle Area; *BMI* Body Mass Index.

**Table 2:** Measurement distribution of the muscle mass index.

Subjects	Variable	Mean	IQR	Quantile				
				0	25	50	75	100
Total	SMA	110.5	38.3	28.5	90.1	107.4	128.5	237.8
	NAMA	52.3	36.3	1.7	32.3	48.1	68.6	174.2
	LAMA	58.2	25.3	2.9	44.5	56.2	69.7	159.2
30-day deceased	SMA	107.7	36.9	28.5	87.5	104	124.3	224.6
	NAMA	49.8	34.7	1.7	30.4	45.3	65.1	167.5
	LAMA	57.9	24.3	2.9	44.3	56	68.6	159.2
30-day survivor	SMA	113.6	39.2	38.5	92.7	110.3	131.9	237.8
	NAMA	55	36.7	3.7	34.1	51.8	70.8	174.2
	LAMA	58.6	26.2	9	44.6	56.3	70.8	140.3

*IQR* interquartile range; *SMA* skeletal muscle area; *NAMA* normal attenuation muscle area; *LAMA* low attenuation muscle area.

**Figure 2:**
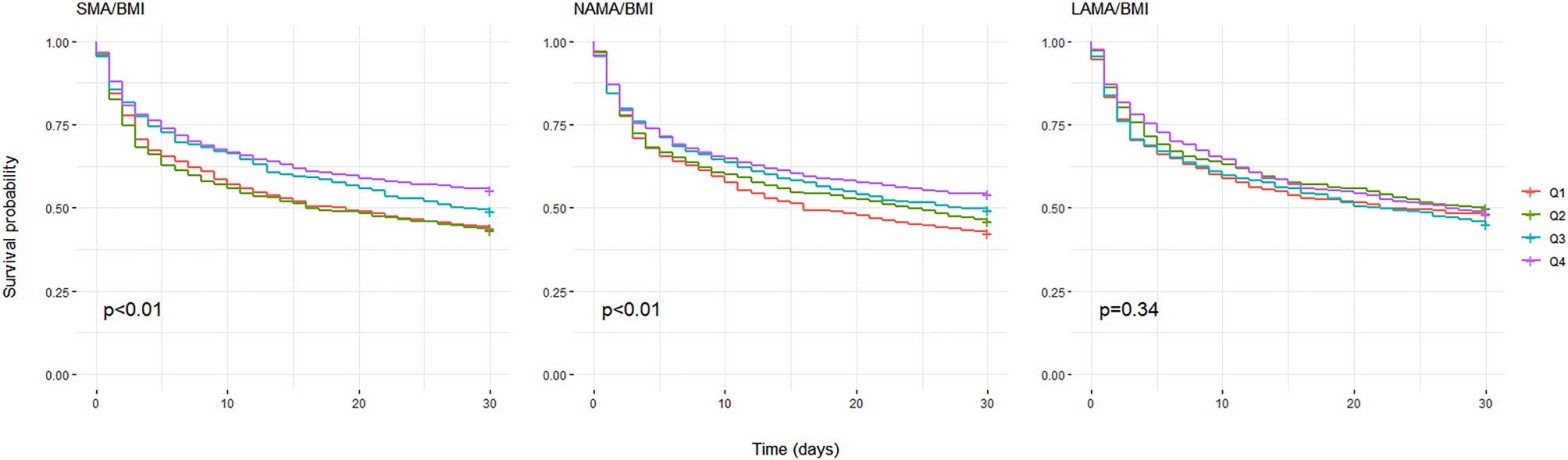
Kaplan–Meier plot between 30-day mortality and muscle mass index.

Figure 2 and Supplementary Fig. S1 show the Kaplan–Meier plot of the 30-day mortality and quartile of the muscle mass index. The survival curve was significantly different according to the quartiles of SMA, SMA/height^2^, SMA/weight, and SMA/BMI, indicating that survival probability was highest in the highest quartile of the SMA-related index. Similar results were found for the NAMA-related index; however, the log-rank test for survival time between LAMA and 30-day mortality was insignificant.

The HRs of 30-day mortality associated with various muscle measurements adjusted for body size are shown in Table 3 and Supplementary Tables S1, S2, and S3. In the crude model, SMA in the highest quartile had an HR of 0.67 (95% CI 0.56–0.79) compared with the lowest quartile. In addition, an IQR (38.3 cm^2^) increase in SMA was associated with decreased mortality risk (HR:0.82, 95% CI:0.76–0.89), and the association between SMA and 30-day mortality remained significant in the fully adjusted model. Moreover, the association between LAMA and 30-day mortality was significant in model 3. An IQR (25.3 cm^2^) increase in LAMA was associated with decreased mortality risk (HR:0.78, 95% CI:0.66–0.92). In addition, HRs were shown as 0.77 (95% CI, 0.66–0.91), 0.73 (95% CI, 0.62–0.85), and 0.74 (95% CI, 0.64–0.87) when adjusted by the square of height, weight, and BMI, respectively. We also found the area under the curve of 0.751 for SMA/BMI, 0.748 for NAMA/BMI, and 0.746 for LAMA/BMI (Suppplemenatary Fig. S2).

**Table 3:** Hazard ratio of 30-day mortality associated with muscle mass index.

Variable	Model 1	Model 2	Model 3
SMA/BMI			
Q1	1 [Reference]	1 [Reference]	1 [Reference]
Q2	1.01 (0.86,1.19)	0.85 (0.60,1.20)	0.77 (0.53,1.11)
Q3	0.83 (0.71,0.99)	0.75 (0.51,1.09)	0.66 (0.44,0.99)
Q4	0.73 (0.61,0.86)	0.66 (0.44,0.98)	0.58 (0.38,0.88)
P for trend	0.01	0.03	0.01
Linear	0.84 (0.78,0.92)	0.84 (0.69,1.01)	0.79 (0.65,0.97)
NAMA /BMI			
Q1	1 [Reference]	1 [Reference]	1 [Reference]
Q2	0.92 (0.78,1.09)	0.69 (0.49,0.98)	0.70 (0.49,1.01)
Q3	0.82 (0.69,0.97)	0.63 (0.44,0.89)	0.67 (0.47,0.97)
Q4	0.74 (0.62,0.88)	0.89 (0.61,1.31)	1.05 (0.71,1.57)
P for trend	< 0.001	0.53	0.8
Linear	0.85 (0.78,0.93)	0.99 (0.82,1.19)	1.07 (0.88,1.31)
LAMA /BMI			
Q1	1 [Reference]	1 [Reference]	1 [Reference]
Q2	0.95 (0.80,1.12)	0.98 (0.71,1.36)	0.98 (0.70,1.37)
Q3	1.06 (0.90,1.25)	0.96 (0.70,1.32)	0.93 (0.67,1.28)
Q4	0.94 (0.79,1.11)	0.81 (0.58,1.11)	0.61 (0.44,0.86)
P for trend	0.78	0.19	0.01
Linear	0.97 (0.90,1.04)	0.84 (0.72,0.97)	0.74 (0.64,0.87)

The table illustrates the hazard ratio of 30-day mortality associated with an increase in skeletal muscle mass (SMA), normal attenuation muscle area (NAMA), and low attenuation muscle area (LAMA) adjusted by BMI in various models.

**Figure 3:**
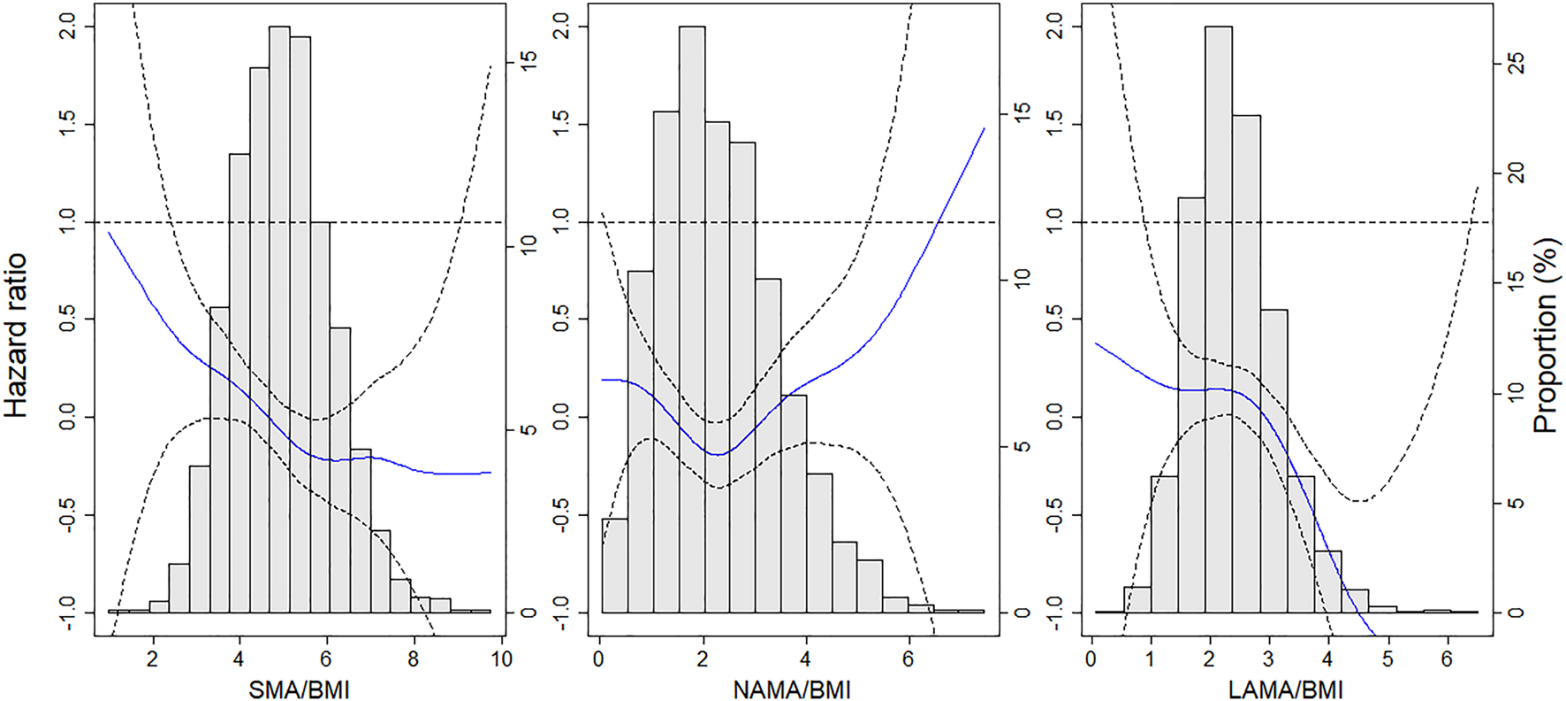
Spline curve of hazard ratio and 95% confidence interval. Spline curve of hazard ratio (blue line) and 95% confidence interval (grey shaded area) associated with SMA/BMI, NAMA/BMI, and LAMA/BMI. *SMA*, skeletal muscle area; *BMI*, body mass index; *NAMA*, normal attenuation muscle area; *LAMA*, low attenuation muscle area.

We found inverse effects of SMA and LAMA on the 30-day mortality in the spline model (Fig. 3 and Supplementary Fig. S3). The dose–response curve seemed to have a threshold for SMA and SMA/height^2^, but the association between SMA/BMI and mortality showed distinct inverse linearity. Further, similar relationships were observed in the effects of LAMA, regardless of height, weight, and BMI adjustments.

**Figure 4:**
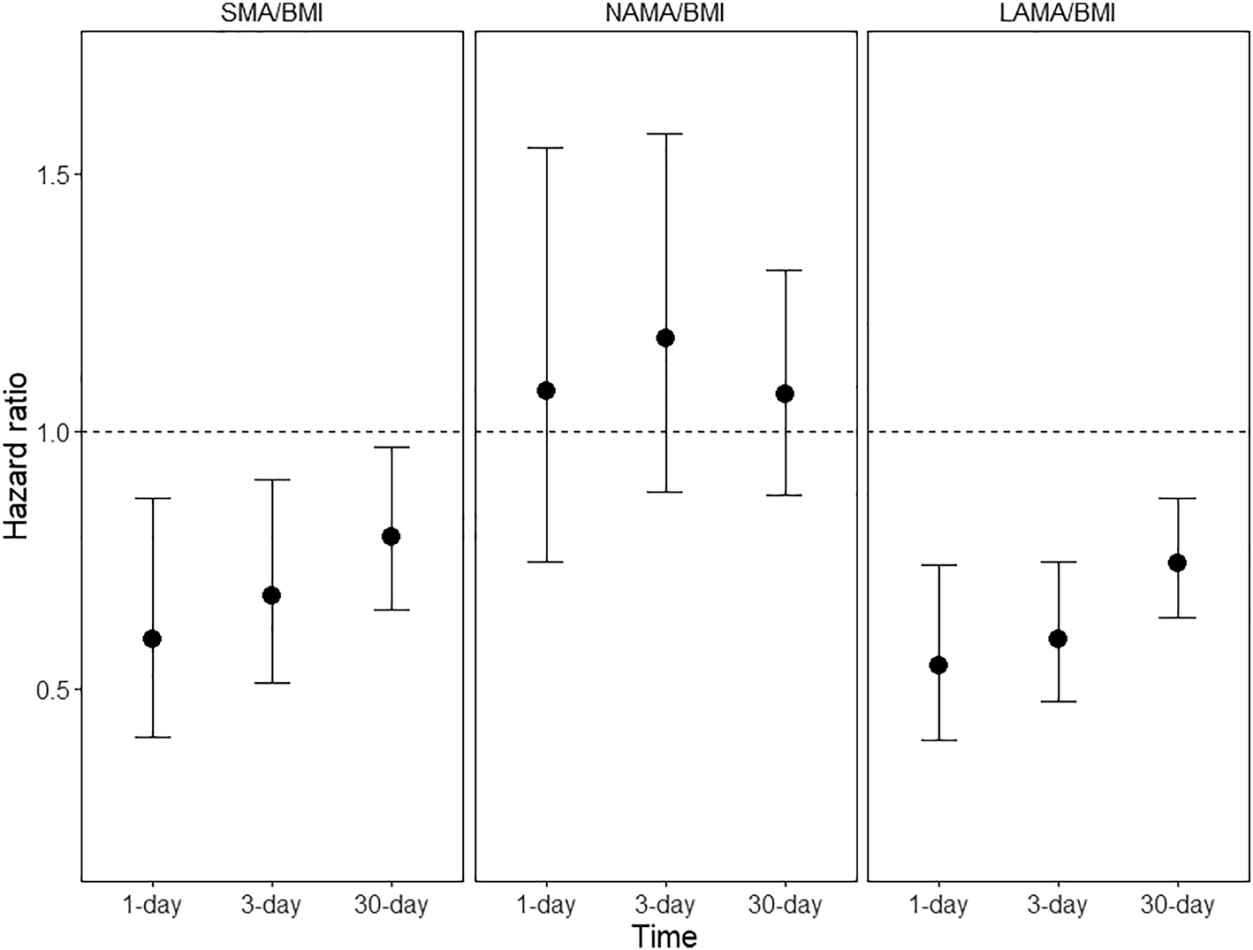
The hazard ratios of muscle index on 1-day, 3-day, and 30-day mortalities in the fully adjusted model. *SMA*, skeletal muscle area; *BMI*, body mass index; *NAMA*, normal attenuation muscle area; *LAMA*, low attenuation muscle area.

We compared the 30-day mortality risk to 1- and 3-day mortality risks associated with various muscle mass indices (Fig. 4 and Supplementary Fig. S4). HRs associated with an increase of SMA/BMI were 0.60 (95% CI, 0.41–0.87) on 1-day mortality, 0.68 (95% CI, 0.51–0.90) on 3-day mortality, and 0.79 (95% CI, 0.65–0.97) on 30-day mortality. In addition, stronger protective effects of LAMA/BMI on 1-day mortality (HR:0.54, 95% CI:0.40–0.74) were observed than on 3-day (HR:0.59, 95% CI:0.47–0.75) and 30-day (HR:0.74, 95% CI:0.64–0.87) mortalities. In contrast, non-significant effects of the NAMA-related index were observed at all-time points of mortality.

Furthermore, stratified analyses of sex, age, APACHE II, and SOFA score were conducted to determine the association between 30-day mortality and muscle mass index adjusted for BMI in a fully adjusted model (Table 4). In sex-specific associations, males had a 23% decreased risk associated with increased SMA/BMI (HR:0.77, 95% CI:0.61–0.96), whereas non-significant effects were found in females (HR:0.95, 95% CI:0.61–1.46). Consistent results were estimated for LAMA/BMI. In addition, those aged ≥ 65 years had protective effects of SMA/BMI (HR:0.71, 95% CI:0.55–0.91) and LAMA/BMI (HR:0.64, 95% CI:0.52–0.78) on mortality. Moreover, lower risks associated with increased LAMA/BMI were observed in the high-score group of APACHE II (HR:0.70, 95% CI:0.53–0.92) and SOFA (HR:0.69, 95% CI:0.48–0.97). In addition, we found an inverse association between LAMA/BMI and mortality in patients without hypertension (HR 0.70, 95% CI 0.59–0.84) and diabetes (HR 0.80, 95% CI 0.65–0.98).

**Table 4:** Stratified association between muscle mass adjusted by BMI and 30-day mortality by sex, age, APACHE score, and SOFA score.

	N	SMA/BMI	NAMA/BMI	LAMA/BMI
Sex				
Male	1318	0.77 (0.61,0.96)	1.03 (0.83,1.29)	0.73 (0.61,0.88)
Female	882	0.95 (0.61,1.46)	1.33 (0.79,2.23)	0.79 (0.56,1.10)
Age				
< 65	933	1.01 (0.72,1.41)	0.98 (0.72,1.34)	1.04 (0.78,1.37)
≥ 65	1267	0.71 (0.55,0.91)	1.14 (0.87,1.49)	0.64 (0.52,0.78)
APACHE II				
< Median	879	0.82 (0.54,1.26)	1.04 (0.67,1.61)	0.76 (0.53,1.10)
≥ Median	1008	0.88 (0.63,1.23)	1.23 (0.89,1.72)	0.70 (0.53,0.92)
SOFA				
< Median	602	0.89 (0.47,1.69)	1.35 (0.71,2.57)	0.59 (0.32,1.08)
≥ Median	704	0.93 (0.61,1.43)	1.28 (0.88,1.87)	0.69 (0.48,0.97)
AKI cause				
Sepsis	905	0.72 (0.47,1.10)	1.16 (0.75,1.78)	0.56 (0.38,0.82)
Non-sepsis	1295	0.90 (0.67,1.21)	1.27 (0.95,1.71)	0.67 (0.52,0.87)
Hypertension				
No	1362	0.86 (0.69,1.07)	1.21 (0.97,1.51)	0.70 (0.59,0.84)
Yes	838	0.75 (0.49,1.14)	0.91 (0.58,1.40)	0.84 (0.62,1.14)
Diabetes				
No	1412	0.77 (0.58,1.03)	1.00 (0.76,1.31)	0.80 (0.65,0.98)
Yes	788	0.56 (0.30,1.04)	0.61 (0.30,1.24)	0.77 (0.48,1.24)

*SMA* skeletal muscle mass; *NAMA* normal attenuation muscle area; *LAMA* low attenuation muscle area; *BMI* body mass index; *APACHE II* Acute Physiology and Chronic Health Evaluation; *SOFA* sequential organ failure assessment; *AKI*, acute kidney injury.

## Discussion

In this large retrospective cohort of patients with AKI who underwent CRRT from multiple medical centers in Korea between 2006 and 2021, the increase in muscle mass measured by validated software using selected L3 levels from CT images was associated with a decreased risk of mortality within 1, 3, and 30 days. Consistent results were obtained when muscle mass was adjusted for height^2^, weight, and BMI. In the analysis of muscle density, LAMA had a significant inverse effect on mortality. In addition, we confirmed the strong protective effects of muscle mass on short-term mortality. Similarly, stronger inverse associations were observed in men, those aged over 65 years, those with high APACHE II and SOFA scores, and patients without hypertension and diabetes.

To the best of our knowledge, this is the first study to evaluate the effects of muscle mass on the mortality of patients with AKI who underwent CRRT. Our findings are consistent with a retrospective cohort study of 226 patients in the ICU between 2008 and 2010, showing that sarcopenia was an independent risk factor for 90-day mortality (OR:1.05, 95% CI:1.03–1.08)^21^. A USA study also reported that an increase in muscle mass measured from the erector spinae muscle at the twelfth thoracic vertebra (T12) was associated with a decreased risk of 6-month mortality (OR:0.96, 95% CI:0.94–0.97) and dependent discharge (OR:0.98, 95% CI:0.96–0.99)^22^. With low muscle mass, chronic inflammatory conditions are induced; the promotion of a catabolic state deteriorates organ functions through pro-inflammatory cytokines such as tumor necrosis factor-alpha (TNF-α), CRP, interleukin-6 (IL-6), and interleukin (IL)-8^23,24^. Dysregulation of hormones, including growth hormone, testosterone, thyroid hormone, and insulin-like growth factor-1 (IGF-1), also promotes protein degradation and suppresses protein synthesis^25,26^. In addition, protein-energy wasting associated with metabolic stress and adverse clinical outcomes is accelerated by hormonal derangement in patients with AKI^27^. Among these mechanisms, patients with low muscle mass are known to have poor prognoses.

In this study, whole-body metrics were represented by a single cross-sectional CT image at L3. Even though whole-body scanning is the most accurate assessment method, single-slice CT or MRI images have been used as practical evaluation methods for muscle mass in the whole body due to their effectiveness in terms of time and cost. However, no standardized protocol has yet been established for level selection. Several previous studies have used different levels to represent the muscle mass at the L4-L5^28^, T12^29,30^, and third cervical vertebrae^31^. However, in most previous studies, single images at L3 derived from CT have been the most common and reliable assessment methods for evaluating skeletal muscle mass^32–34^.

We found more protective effects of LAMA on mortality than NAMA, indicating that muscle mass was a determining factor in the mortality of patients in the ICU, even when the muscle density was low. In our results, the average LAMA (58.2 cm^2^) in all patients was higher than the average NAMA (52.3 cm^2^), and a significant difference in the mean by t-test was shown (p < 0.001). A similar result was found in a study that assessed the effects of muscle quality on the risk of a metabolically unhealthy phenotype characterized by the presence of metabolic syndrome, hypertension, and diabetes^18^. They discovered that people with obesity had more poor-quality muscles estimated at the L3 level than healthy individuals. Another study also reported that obese adult individuals had a 75–92% prevalence of poor muscle quality, defined by strength and power^35^. However, inconsistent results were shown in a study suggesting that cardiovascular disease in patients with type 2 diabetes was associated with low arm muscle quality, which was defined as muscle strength (kg) divided by arm muscle mass (kg), and high visceral fat accumulation estimated by bioelectrical impedance analysis (BIA) (OR:2.72, 95% CI:1.19–6.61)^36^. In addition, reduction in muscle quality assessed by hand grip strength was an important risk factor for increased BMI and fat mass among adults aged 40–59 years^37^. The inconsistent relationship between low muscle quality and health outcomes in AKI patients undergoing CRRT may be due to the rapid loss of muscle mass during treatment, which may mask the prognostic contribution of NAMA and potentially inflate the effect of a high proportion of LAMA. Despite this, there is limited research on the prognostic significance of muscle quality and the underlying mechanisms. Therefore, further investigation is warranted to clarify the association and better understand the mechanisms involved.

A stronger protective effect of muscle mass index on 30-day mortality was discovered in males, those aged over 65 years, and the high APACHE II and SOFA score group. Inconsistent results in sex-specific associations have been reported in previous studies. Sarcopenia was a risk factor for mortality in both male (HR:2.46, 95% CI:1.86–3.25) and female (HR:2.16, 95% CI:1.24–3.78) patients with cirrhosis^38^. In addition, women had protective effects of higher muscle mass assessed using dual-energy X-ray absorptiometry (DXA) to reduce cardiovascular mortality in adults in the USA^39^. However, our findings are consistent with those of subgroup studies on age. Further, a study that investigated the effects of SMA on mortality of older patients with sepsis showed a significant association in both the 60–80 years (OR:0.96, 95% CI 0.92–0.99) and over 80 years groups (OR:0.89, 95% CI:0.81–0.98)^40^. In addition, we found the protective effects of muscle in the high score group of APACHE II and SOFA, indicating that those with high severity in the ICU had a survival benefit from muscle on mortality.

Our study had some limitations. First, there might have been incorrect selection of L3 levels from CT images, although experts in the field extracted the inferior endplate of the L3 images. However, the highest correlations with muscle mass in the total body were estimated in the skeletal muscle area 5 cm above the L4-L5 levels^41^. In addition, L2, L4, and L5 images could be used as alternative levels for measuring muscles^42^. Second, we estimated SMA in slice CT images; however, DXA or BIA was recommended for measuring appendicular skeletal muscle mass to define sarcopenia in the AWGS, and measurement using CT is an alternative method that considers the characteristics of critically ill patients to estimate SMA. Third, owing to the limitations of retrospective data collection, the possibility that only patients who underwent CT for diagnostic evaluation may have induced biased results cannot be excluded. Fourth, since we retrospectively collected cases in which clinicians at each institution independently judged that CRRT was necessary in each case without using any specific shared criteria for CRRT initiation, it is possible that the study population was heterogeneous.

Despite these limitations, this study has some advantages. First, our study confirmed the adverse effects of low muscle mass on mortality in critically ill patients in a large retrospective cohort of patients with AKI requiring CRRT in multiple medical centers. Second, automated AI software was applied to estimate the muscle mass and density obtained from a single slice at the L3 level from CT images. Third, we estimated the effects of muscle mass and muscle density adjusted by body size on mortality and found a significant inverse linear relationship between mortality associated with SMA and LAMA. Finally, stratified analyses by sex, age, and severity scoring were conducted to investigate the groups susceptible to muscle mass index.

## Conclusions

Our findings suggest that even if the muscle quality is low, muscle mass can be a determining factor of mortality in critically ill patients and can be a useful index for clinical mortality evaluation in patients with severe AKI.

## Supplementary Information

Supplementary Information.

## Data Availability

The datasets used during the current study are available from the corresponding author on reasonable request.

## References

[1] Wang HE, Muntner P, Chertow GM, Warnock DG (2012). Acute kidney injury and mortality in hospitalized patients. Am. J. Nephrol..

[2] Levy EM, Viscoli CM, Horwitz RI (1996). The effect of acute renal failure on mortality. A cohort analysis. JAMA.

[3] Hyman A, Mendelssohn DC (2002). Current Canadian approaches to dialysis for acute renal failure in the icu. Am. J. Nephrol..

[4] Chertow GM, Burdick E, Honour M, Bonventre JV, Bates DW (2005). Acute kidney injury, mortality, length of stay, and costs in hospitalized patients. J. Am. Soc. Nephrol..

[5] Uchino S (2005). Acute renal failure in critically ill patients: A multinational, multicenter study. JAMA.

[6] Landi F (2012). Sarcopenia as a risk factor for falls in elderly individuals: Results from the ilsirente study. Clin. Nutr..

[7] Rosenberg IH (1997). Sarcopenia: Origins and clinical relevance. J. Nutr..

[8] Maeda K, Akagi J (2016). Sarcopenia is an independent risk factor of dysphagia in hospitalized older people. Geriatr. Gerontol. Int..

[9] Ravussin E, Lillioja S, Anderson TE, Christin L, Bogardus C (1986). Determinants of 24-hour energy expenditure in man. Methods and results using a respiratory chamber. J. Clin. Invest..

[10] Chen LK (2020). Asian working group for sarcopenia: 2019 consensus update on sarcopenia diagnosis and treatment. J. Am. Med. Dir. Assoc..

[11] Han JS (2020). Association of body composition with long-term survival in non-metastatic rectal cancer patients. Cancer Res. Treat..

[12] Kim DW (2020). Assessment of myosteatosis on computed tomography by automatic generation of a muscle quality map using a web-based toolkit: Feasibility study. JMIR Med. Inform..

[13] Charlson ME, Pompei P, Ales KL, MacKenzie CR (1987). A new method of classifying prognostic comorbidity in longitudinal studies: Development and validation. J. Chronic Dis..

[14] Knaus WA, Draper EA, Wagner DP, Zimmerman JE (1985). Apache ii: A severity of disease classification system. Crit. Care Med..

[15] Vincent JL (1996). The sofa (sepsis-related organ failure assessment) score to describe organ dysfunction/failure. On behalf of the working group on sepsis-related problems of the European society of intensive care medicine. Intensive Care Med..

[16] Gomez-Perez SL (2016). Measuring abdominal circumference and skeletal muscle from a single cross-sectional computed tomography image: A step-by-step guide for clinicians using National Institutes of Health ImageJ. JPEN J. Parenter. Enteral Nutr..

[17] Ha J (2021). Development of a fully automatic deep learning system for l3 selection and body composition assessment on computed tomography. Sci. Rep..

[18] Kim EH (2021). Reference data and t-scores of lumbar skeletal muscle area and its skeletal muscle indices measured by CT scan in a healthy Korean population. J. Gerontol. A Biol. Sci. Med. Sci..

[19] Park HJ (2020). Development and validation of a deep learning system for segmentation of abdominal muscle and fat on computed tomography. Korean J. Radiol..

[20] Ko Y (2022). Change of computed tomography-based body composition after adrenalectomy in patients with pheochromocytoma. Cancers.

[21] Kashani KB (2017). Evaluating muscle mass by using markers of kidney function: Development of the sarcopenia index. Crit. Care Med..

[22] Jaitovich A (2020). ICU admission body composition: Skeletal muscle, bone, and fat effects on mortality and disability at hospital discharge—a prospective, cohort study. Crit. Care.

[23] Curcio F (2016). Biomarkers in sarcopenia: A multifactorial approach. Exp. Gerontol..

[24] Schaap LA (2009). Higher inflammatory marker levels in older persons: Associations with 5-year change in muscle mass and muscle strength. J. Gerontol. A Biol. Sci. Med. Sci..

[25] Frost RA, Lang CH (2012). Multifaceted role of insulin-like growth factors and mammalian target of rapamycin in skeletal muscle. Endocrinol. Metab. Clin. North Am..

[26] Hardy RS (2018). 11 Beta-hydroxysteroid dehydrogenase type 1 regulates synovitis, joint destruction, and systemic bone loss in chronic polyarthritis. J. Autoimmun..

[27] Fouque D (2008). A proposed nomenclature and diagnostic criteria for protein–energy wasting in acute and chronic kidney disease. Kidney Int..

[28] Morrell GR (2016). Psoas muscle cross-sectional area as a measure of whole-body lean muscle mass in maintenance hemodialysis patients. J. Ren. Nutr..

[29] Lenchik L (2021). Automated muscle measurement on chest ct predicts all-cause mortality in older adults from the national lung screening trial. J. Gerontol. A Biol. Sci. Med. Sci..

[30] Tan L (2021). Diagnosing sarcopenia and myosteatosis based on chest computed tomography images in healthy Chinese adults. Insights Imaging.

[31] Zwart AT (2019). CT-measured skeletal muscle mass used to assess frailty in patients with head and neck cancer. J. Cachexia Sarcopenia Muscle.

[32] Derstine BA (2021). Optimal body size adjustment of l3 ct skeletal muscle area for sarcopenia assessment. Sci. Rep..

[33] Liu X (2021). The correlation between skeletal muscle index of the l3 vertebral body and malnutrition in patients with advanced lung cancer. BMC Cancer.

[34] Wang S (2020). The value of l3 skeletal muscle index in evaluating preoperative nutritional risk and long-term prognosis in colorectal cancer patients. Sci. Rep..

[35] Valenzuela PL, Maffiuletti NA, Tringali G, De Col A, Sartorio A (2020). Obesity-associated poor muscle quality: Prevalence and association with age, sex, and body mass index. BMC Musculoskelet. Disord..

[36] Murai J (2018). Low muscle quality in Japanese type 2 diabetic patients with visceral fat accumulation. Cardiovasc. Diabetol..

[37] Silva TLD, Mulder AP (2021). Sarcopenia and poor muscle quality associated with severe obesity in young adults and middle-aged adults. Clin. Nutr. ESPEN.

[38] Tantai X (2022). Effect of sarcopenia on survival in patients with cirrhosis: A meta-analysis. J. Hepatol..

[39] Srikanthan P, Horwich TB, Calfon Press M, Gornbein J, Watson KE (2021). Sex differences in the association of body composition and cardiovascular mortality. J. Am. Heart Assoc..

[40] Shibahashi K, Sugiyama K, Kashiura M, Hamabe Y (2017). Decreasing skeletal muscle as a risk factor for mortality in elderly patients with sepsis: A retrospective cohort study. J. Intensive Care.

[41] Shen W (2004). Total body skeletal muscle and adipose tissue volumes: Estimation from a single abdominal cross-sectional image. J. Appl. Physiol..

[42] Derstine BA (2018). Skeletal muscle cutoff values for sarcopenia diagnosis using t10 to l5 measurements in a healthy us population. Sci. Rep..

