# Supplementary material for: The effects of muscle mass and quality on mortality of patients with acute kidney injury requiring continuous renal replacement therapy
Source: Sci Rep. 2023 May 5;13:7311. doi: 10.1038/s41598-023-33716-9 (PMC10162987; doi:10.1038/s41598-023-33716-9)
Supplement: Supplementary file 1 — Supplementary Information. [file 41598_2023_33716_MOESM1_ESM.docx]

**Additional file for**

**The effects of muscle mass and quality on mortality of patients with Acute Kidney Injury requiring Continuous Renal Replacement Therapy**

Jiyun Jung^1,2,¶^, Jangwook Lee^2,3,¶^, Jeong-Hoon Lim^4^, Yong Chul Kim^5^, Tae Hyun Ban^6^, Woo Yeong Park^7^, Kyeong Min Kim^8^, Kipyo Kim^9^, Sung Woo Lee^10^, Sung Joon Shin^2,3,11^, Seung Seok Han^5^, Dong Ki Kim^5^, Yousun Ko^12^, Kyung Won Kim^13^, Hyosang Kim^14,#,*^, Jae Yoon Park^2,3,11,#,*^

This file includes:

- Supplementary Table S1. The hazard ratio of 30-day mortality associated with increase of skeletal muscle mass in various models
- Supplementary Table S2. The hazard ratio of 30-day mortality associated with increase of normal attenuation muscle area (cm^2^) in various models
- Supplementary Table S3. The hazard ratio of 30-day mortality associated with increase of low attenuation muscle area (cm^2^) in various models
- Supplementary Fig. S1. Kaplan-Meier plot between 30-day mortality and muscle mass index.
- Supplementary Fig. S2. Receiver Operating Characteristic (ROC) curve for predicting the mortality risk associated with muscle index
- Supplementary Fig. S3. Spline curve of hazard ratio (blue line) and 95% confidence interval (grey shaded area) associated with various muscle index
- Supplementary Fig. S4. The hazard ratios of muscle index adjusted by body size on 1-day, 3-day, and 30-day mortality in fully adjusted model.

Supplementary Table S1. The hazard ratio of 30-day mortality associated with increase of skeletal muscle mass in various models

| Variable |  | Model1 | Model2 | Model3 |
| --- | --- | --- | --- | --- |
| SMA (cm^2^) | |  |  |  |
|  | Q1 | 1 [Reference] | 1 [Reference] | 1 [Reference] |
|  | Q2 | 0.91 (0.78,1.07) | 0.89 (0.64,1.23) | 0.88 (0.62,1.23) |
|  | Q3 | 0.81 (0.69,0.96) | 0.75 (0.53,1.05) | 0.73 (0.51,1.05) |
|  | Q4 | 0.67 (0.56,0.79) | 0.66 (0.45,0.96) | 0.67 (0.45,1.00) |
|  | P for trend | 0.01 | 0.02 | 0.03 |
|  | Linear | 0.82 (0.76,0.89) | 0.79 (0.65,0.95) | 0.81 (0.67,1.00) |
| SMA/height^2^ (cm^2^/m^2^) | |  |  |  |
|  | Q1 | 1 [Reference] | 1 [Reference] | 1 [Reference] |
|  | Q2 | 0.82 (0.70,0.97) | 0.66 (0.48,0.91) | 0.65 (0.47,0.91) |
|  | Q3 | 0.69 (0.58,0.81) | 0.55 (0.40,0.76) | 0.56 (0.40,0.78) |
|  | Q4 | 0.60 (0.51,0.71) | 0.63 (0.45,0.87) | 0.66 (0.47,0.92) |
|  | P for trend | 0.01 | 0.01 | 0.01 |
|  | Linear | 0.80 (0.74,0.87) | 0.80 (0.67,0.94) | 0.82 (0.69,0.97) |
| SMA/weight (cm^2^/kg) | |  |  |  |
|  | Q1 | 1 [Reference] | 1 [Reference] | 1 [Reference] |
|  | Q2 | 0.80 (0.68,0.94) | 0.74 (0.53,1.04) | 0.70 (0.49,0.99) |
|  | Q3 | 0.81 (0.69,0.95) | 0.84 (0.61,1.15) | 0.78 (0.56,1.10) |
|  | Q4 | 0.60 (0.51,0.72) | 0.59 (0.42,0.83) | 0.55 (0.38,0.78) |
|  | P for trend | 0.01 | 0.01 | 0.01 |
|  | Linear | 0.83 (0.77,0.90) | 0.84 (0.71,0.98) | 0.80 (0.68,0.94) |

Supplementary Table S2. The hazard ratio of 30-day mortality associated with increase of normal attenuation muscle area (cm^2^) in various models

| Variable |  | Model1 | Model2 | Model3 |
| --- | --- | --- | --- | --- |
| NAMA (cm^2^) | |  |  |  |
|  | Q1 | 1 [Reference] | 1 [Reference] | 1 [Reference] |
|  | Q2 | 0.96 (0.82,1.13) | 0.75 (0.54,1.05) | 0.77 (0.55,1.10) |
|  | Q3 | 0.77 (0.65,0.91) | 0.59 (0.42,0.84) | 0.68 (0.47,0.98) |
|  | Q4 | 0.74 (0.62,0.88) | 0.83 (0.57,1.22) | 1.05 (0.70,1.56) |
|  | P for trend | 0.01 | 0.2 | 0.99 |
|  | Linear | 0.84 (0.77,0.91) | 0.93 (0.77,1.13) | 1.06 (0.86,1.31) |
| NAMA /height^2^ (cm^2^/m^2^) | |  |  |  |
|  | Q1 | 1 [Reference] | 1 [Reference] | 1 [Reference] |
|  | Q2 | 0.93 (0.79,1.10) | 0.74 (0.54,1.03) | 0.73 (0.52,1.03) |
|  | Q3 | 0.76 (0.64,0.90) | 0.67 (0.48,0.94) | 0.74 (0.52,1.05) |
|  | Q4 | 0.72 (0.60,0.86) | 0.80 (0.56,1.14) | 1.00 (0.68,1.46) |
|  | P for trend | 0.01 | 0.19 | 0.99 |
|  | Linear | 0.83 (0.76,0.90) | 0.93 (0.77,1.11) | 1.05 (0.86,1.28) |
| NAMA /weight (cm^2^/kg) | |  |  |  |
|  | Q1 | 1 [Reference] | 1 [Reference] | 1 [Reference] |
|  | Q2 | 0.96 (0.82,1.13) | 0.70 (0.50,0.97) | 0.70 (0.50,0.99) |
|  | Q3 | 0.77 (0.65,0.91) | 0.59 (0.42,0.83) | 0.63 (0.44,0.90) |
|  | Q4 | 0.76 (0.64,0.91) | 0.83 (0.59,1.17) | 0.97 (0.67,1.39) |
|  | P for trend | 0.01 | 0.23 | 0.76 |
|  | Linear | 0.85 (0.78,0.92) | 0.98 (0.82,1.17) | 1.06 (0.87,1.28) |

Supplementary Table S3. The hazard ratio of 30-day mortality associated with increase of low attenuation muscle area (cm^2^) in various models

| Variable |  | Model1 | Model2 | Model3 |
| --- | --- | --- | --- | --- |
| LAMA (cm^2^) | |  |  |  |
|  | Q1 | 1 [Reference] | 1 [Reference] | 1 [Reference] |
|  | Q2 | 0.96 (0.81,1.13) | 0.98 (0.70,1.37) | 0.99 (0.69,1.40) |
|  | Q3 | 1.01 (0.86,1.19) | 1.01 (0.74,1.37) | 0.88 (0.63,1.21) |
|  | Q4 | 0.83 (0.70,0.99) | 0.69 (0.50,0.95) | 0.61 (0.44,0.85) |
|  | P for trend | 0.08 | 0.03 | 0.01 |
|  | Linear | 0.95 (0.87,1.03) | 0.84 (0.72,0.98) | 0.78 (0.66,0.92) |
| LAMA /height^2^ (cm^2^/m^2^) | |  |  |  |
|  | Q1 | 1 [Reference] | 1 [Reference] | 1 [Reference] |
|  | Q2 | 0.93 (0.79,1.10) | 0.92 (0.68,1.27) | 0.94 (0.67,1.30) |
|  | Q3 | 0.95 (0.81,1.12) | 0.85 (0.63,1.16) | 0.75 (0.55,1.04) |
|  | Q4 | 0.84 (0.71,1.00) | 0.65 (0.47,0.89) | 0.56 (0.40,0.79) |
|  | P for trend | 0.08 | 0.01 | 0.01 |
|  | Linear | 0.94 (0.87,1.02) | 0.83 (0.71,0.97) | 0.77 (0.66,0.91) |
| LAMA /weight (cm^2^/kg) | |  |  |  |
|  | Q1 | 1 [Reference] | 1 [Reference] | 1 [Reference] |
|  | Q2 | 1.02 (0.86,1.20) | 1.18 (0.86,1.64) | 1.19 (0.85,1.66) |
|  | Q3 | 0.98 (0.83,1.16) | 0.96 (0.70,1.31) | 0.88 (0.63,1.23) |
|  | Q4 | 0.92 (0.78,1.10) | 0.78 (0.56,1.09) | 0.60 (0.42,0.85) |
|  | P for trend | 0.31 | 0.05 | 0.01 |
|  | Linear | 0.96 (0.89,1.04) | 0.82 (0.71,0.95) | 0.73 (0.62,0.85) |


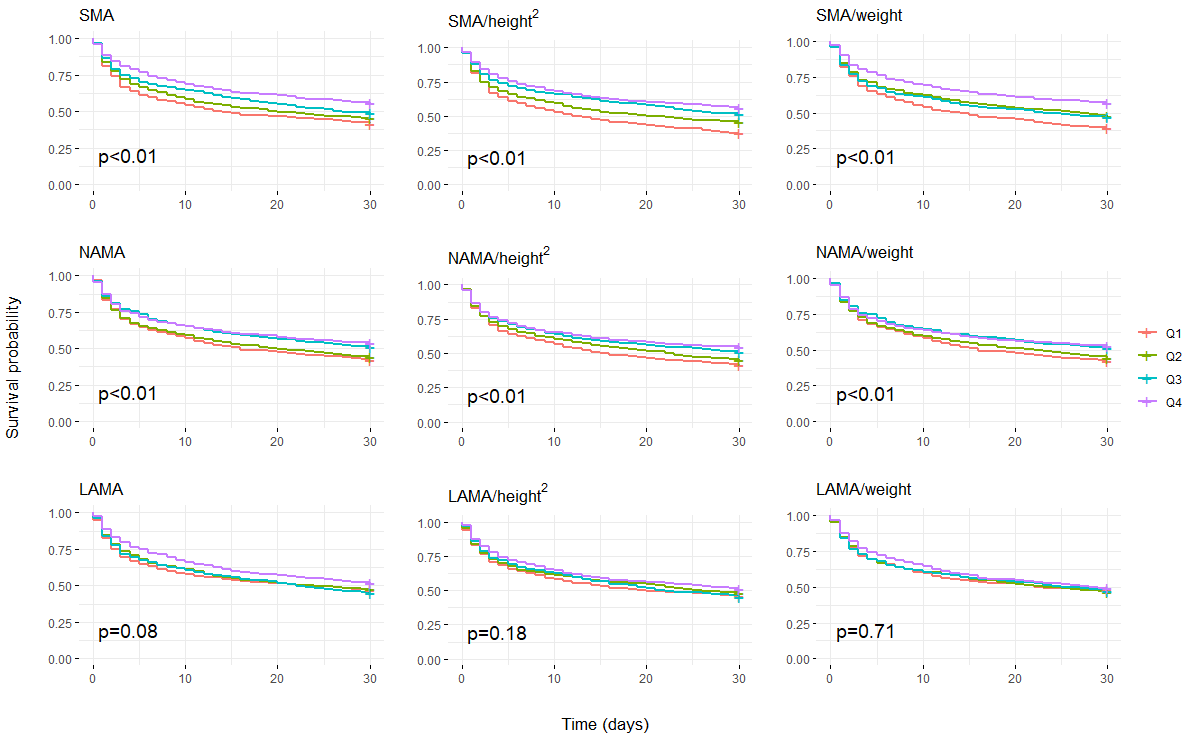
 Supplementary Fig. S1. Kaplan-Meier plot between 30-day mortality and muscle mass index.


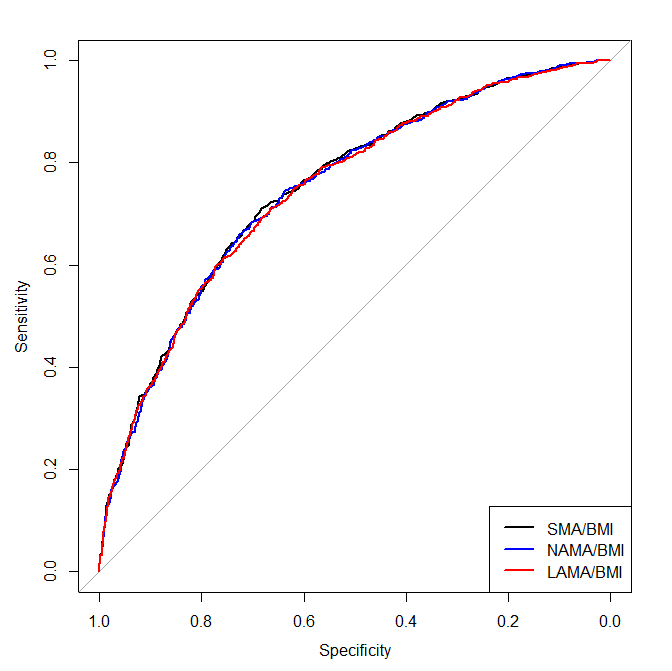


Supplementary Fig. S2. Receiver Operating Characteristic (ROC) curve for predicting the mortality risk associated with muscle index


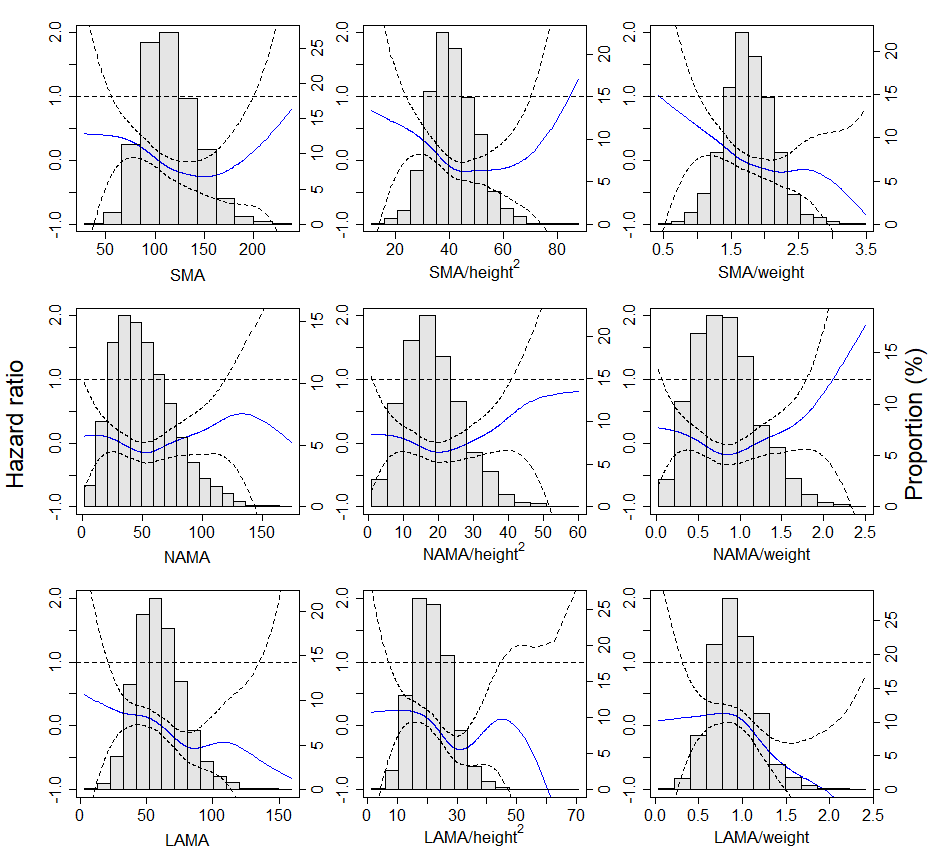


Supplementary Fig. S3. Spline curve of hazard ratio (blue line) and 95% confidence interval (grey shaded area) associated with various muscle index


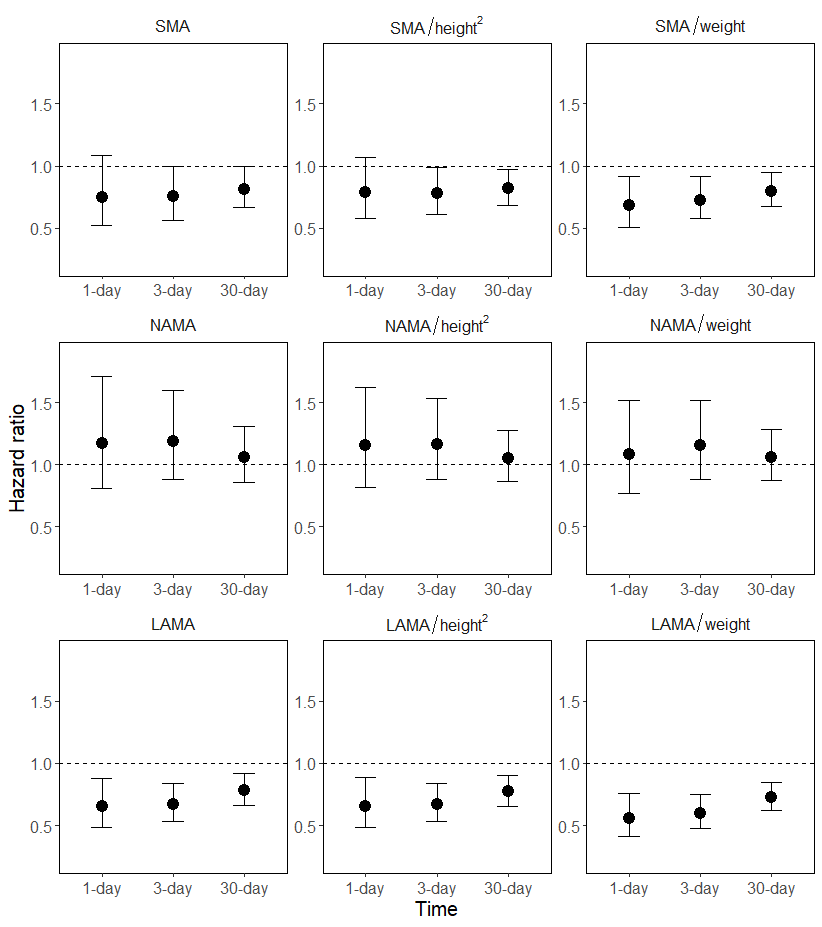


Supplementary Fig. S4. The hazard ratios of muscle index adjusted by body size on 1-day, 3-day, and 30-day mortality in fully adjusted model.
